# ECO: A Generic Eutrophication Model Including Comprehensive Sediment-Water Interaction

**DOI:** 10.1371/journal.pone.0068104

**Published:** 2013-07-03

**Authors:** Johannes G. C. Smits, Jan K. L. van Beek

**Affiliations:** Marine and Coastal Systems, Deltares, Delft, The Netherlands; Dowling College, United States of America

## Abstract

The content and calibration of the comprehensive generic 3D eutrophication model ECO for water and sediment quality is presented. Based on a computational grid for water and sediment, ECO is used as a tool for water quality management to simulate concentrations and mass fluxes of nutrients (N, P, Si), phytoplankton species, detrital organic matter, electron acceptors and related substances. ECO combines integral simulation of water and sediment quality with sediment diagenesis and closed mass balances. Its advanced process formulations for substances in the water column and the bed sediment were developed to allow for a much more dynamic calculation of the sediment-water exchange fluxes of nutrients as resulting from steep concentration gradients across the sediment-water interface than is possible with other eutrophication models. ECO is to more accurately calculate the accumulation of organic matter and nutrients in the sediment, and to allow for more accurate prediction of phytoplankton biomass and water quality in response to mitigative measures such as nutrient load reduction. ECO was calibrated for shallow Lake Veluwe (The Netherlands). Due to restoration measures this lake underwent a transition from hypertrophic conditions to moderately eutrophic conditions, leading to the extensive colonization by submerged macrophytes. ECO reproduces observed water quality well for the transition period of ten years. The values of its process coefficients are in line with ranges derived from literature. ECO’s calculation results underline the importance of redox processes and phosphate speciation for the nutrient return fluxes. Among other things, the results suggest that authigenic formation of a stable apatite-like mineral in the sediment can contribute significantly to oligotrophication of a lake after a phosphorus load reduction.

## Introduction

Eutrophication models tend to focus on processes in the water column. The benthic-pelagic coupling and processes in the sediment have been neglected or very much simplified in most models [1]–[8]. However, the longer the residence time in a water system, the more return fluxes of nutrients from the sediment contribute to the availability of nutrients for algae. Because of longer residence times the adequate modeling of return fluxes is generally more important for lakes than for coastal seas. This holds in particular for stratified lakes and reservoirs, in which transient hypoxia affects the return fluxes strongly [9]. Jeppesen et al. [10] argued on the basis of data for 35 lakes that due to internal loading P oligotrophication is delayed 5 to 10 years after external load reduction.

The modeling of benthic-pelagic coupling requires the incorporation of sediment diagenesis processes. Sediment diagenesis models were developed to study processes or to quantify exchange fluxes between sediment and overlying water [11]–[13]. Generic diagenesis models are dynamic and multi-layered. Apart from organic matter and nutrients, these models contain C-Fe-Mn-S-Ca chemistry [14]–[20]. Although algal primary production in the water column often formed the broader context, sediment diagenesis models were mostly used with observed water quality as forcing function. Reducing the computational burden, simplified sediment sub-models were developed for incorporation in eutrophication models as process modules, for marine systems [21]–[24], [13] as well as for freshwater systems [25]–[28]. These models were used to study nutrient budgets [4], [17], eutrophication level [2], [8], the effects of hypoxia [29], [30] and the effects of mitigative measures [5], [6], [26], [31]. Many of these models are deficient as to specific processes, or cover only one or two of the nutrients (N, P, Si). The simplified substances and processes content of their sediment sub-models often resulted in insufficient temporal variability of the nutrient return fluxes.

The substances usually included in eutrophication models concern the biomasses of phytoplankton species, one or two particulate detrital organic matter fractions and dissolved inorganic nutrients (N, P,Si). Inorganic suspended matter is often included, because it contributes to light limitation of phytoplankton. For the enhanced modeling of phytoplankton, water quality and nutrient return fluxes the addition of some of the following substances is required. Dissolved organic matter strongly affects the under water light regime. Oxygen, nitrate, manganese(IV), iron(III), sulphate and methane participate in redox processes that affect the speciation of phosphate in the sediment. The adsorption of phosphate to iron(III) oxyhydroxides and the precipitation of phosphate minerals determine the concentration of dissolved phosphate. The dissolution of opal silicate rules the concentration of dissolved silicate.

For the formulation of the diagenetic processes in eutrophication models various kinetic concepts are available that differ with regard to reaction mechanism and details taken into account [11], [12]. Simplified models mostly use first order kinetics, the process rate being proportional to the concentration of the main reactant. The multi-G-model for the decomposition of organic matter [32] is an example of first-order kinetics. Advanced models may contain more versatile Michaelis-Menten kinetics, that take into account the maximum rate and the inhibition of a microbial enzymatic process [22]. The kinetics for mineral formation take the difference between actual and equilibrium conditions as a driving force, based on a pH-dependent concentration or a solubility product [14]. Theoretically, the more comprehensive a model is and the more realistic its kinetics are, the more generic it may be, because it predicts process rates reliably for a wider range of conditions. However, trade-offs need to be made to minimize the number of poorly quantifiable parameters.

In this article we present the 3D eutrophication model ECO, aiming at the comprehensive, deterministic and accurate simulation of phytoplankton biomass, water quality, sediment diagenesis and sediment-water interaction. We developed its advanced process formulations for substances in the water column and the bed sediment to allow for a much more dynamic calculation of the sediment-water exchange fluxes of nutrients (N, P, Si) as resulting from steep concentration gradients across the sediment-water interface than is possible with other eutrophication models, including its predecessor model DBS [25], [26]. ECO is to more deterministically and consequently more accurately calculate the accumulation of organic matter and nutrients in the sediment, and to allow for more accurate prediction of phytoplankton biomass and water quality in response to mitigative measures such as nutrient load reduction. Its generic process formulations should make ECO applicable to both freshwater and marine systems. Due to its modular set-up ECO has minimal restrictions with regard to the simulation of spatial and temporal variability and the modification of its substances and processes content. The calibration of the ECO model for Lake Veluwe in the Netherlands is described. The resulting values of process coefficients are compared with ranges of reported values. The simulation results of the calibrated model, many of which are additional to the results of predecessor model DBS [25], [26], are discussed. These results underline the importance of redox processes and phosphate speciation for the nutrient return fluxes, and entail new conclusions concerning the mechanisms that led to the oligotrophication of Lake Veluwe.

## Description of the Model ECO

### Structure, Schematization, Mass Transport

ECO is based on the open source water quality modeling framework D-Water Quality (DELWAQ), that facilitates the selection of substances and processes from a processes library and has many options for mass conservative numerical integration [24], [33]. A DELWAQ model is defined by means of an input file with a system definition (substances, processes and pertinent coefficients), a computational grid, an initial composition, timers, flow fields (a dynamic water balance), dispersion coefficients, loads and meteorological forcing. All input parameters in the model can be specified as constants or temporally and/or spatially varying parameters. The output of a DELWAQ model includes the concentrations and mass balances of all simulated substances, the magnitudes of all imposed and simulated parameters, and the mass fluxes of all simulated processes.

DELWAQ solves the following 3D advection diffusion reaction equation for a finite volumes computational grid:
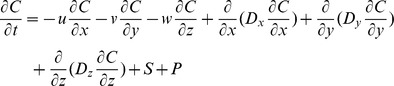
where

C: concentration (g.m^−3^)

u, v, w: components of the velocity vector (m.s^−1^)

Dx, Dy, Dz: components of the dispersion tensor (m^2^.s^−1^)

x, y, z: coordinates in three spatial dimensions (m)

S: sources and sinks of mass due to loads and boundaries (g.m^−3^.s^−1^)

P: sources and sinks of mass due to processes (g.m^−3^.s^−1^)

t: time (s)

ECO dynamically simulates a set of substances and processes on a computational grid that is composed of a water grid and a sediment grid having the same horizontal resolution, allowing for a much finer vertical resolution than DBS [25] and a spatial detailedness that is innovative for eutrophication models. Each water compartment has a sediment column, consisting of a number of layers the thicknesses of which may increase with depth. Thin upper sediment layers allow for a realistic representation of the steep concentration gradients across the sediment-water interface. The number and size of the grid cells is only limited by the maximum acceptable computational burden. The numerical solver applied in the ECO model of Lake Veluwe concerns an explicit upwind scheme in the horizontal direction, combined with an implicit in time scheme with central discretization of advection in the vertical direction. The computational time step is 5 minutes. It was verified that a further reduction of the time step did not affect the simulation results of the Lake Veluwe model significantly. The vertical mass transport in the sediment and across the sediment-water interface resulting from settling, resuspension, dispersion and seepage is generated by a process included in the processes library. This transport leads to the burial of substances below the boundary of the model in the sediment or the addition of substances from below this boundary. Dispersion coefficients and seepage velocity are input parameters. In the Lake Veluwe model, transport in the sediment is based on constant layer thickness and porosity, and on net settling (no resuspension).

### Substances

ECO as applied for Lake Veluwe simulates phytoplankton biomass (C/N/P/Si/S) for five species (ALG_i_), five fractions of detrital organic carbon, nitrogen, phosphorus and sulphur (particulate POC/N/P/S1, POC/N/P/S2, POC/N/P/S3, POC/N/P/S4 and dissolved DOC/N/P/S), nitrate (NO3), ammonium (NH4), dissolved phosphate (PO4), adsorbed phosphate (AAP), vivianite-P (VIVP), apatite-P (APATP), dissolved silicate (Si), opal silicate (OPAL), dissolved oxygen (DO), sulphate (SO4), dissolved and particulate sulphide (SUD, SUP), methane (CH4), two fractions of inorganic matter (IM1, IM2) and chloride (Cl). Compared to DBS [26], POX3, POX4, DOX, APATP, OPAL, all sulphur components, CH4, IM1 and IM2 have been added. Oxygen, sulphate and methane play a dominant role in the decomposition of organic matter, and together with nitrate determine the vertical gradient of the redox potential in the sediment. This gradient regulates the adsorption or desorption of phosphate to iron(III) oxyhydroxides. VIVP and APATP are phosphate minerals that contain iron or calcium, and in sediment are associated with other iron or calcium minerals. OPAL represents the silicate skeletons resulting from diatom mortality. IM1 and IM2 represent silt (<63 µm) and sand (≥63 µm), and are used to fix sediment porosity, to generate burial and to calculate the adsorption capacity for phosphate in the sediment. IM3 is used to force the inorganic suspended sediment concentration, which contributes to the extinction of light and determines the adsorption capacity for phosphate in the water column. Conservative substance Cl was used to check the water balance of the Lake Veluwe model as to accurateness.

### Processes Included in ECO

The processes in ECO are selected from DELWAQ’s processes library. The processes included in ECO for Lake Veluwe are:

- growth, mortality, grazing of phytoplankton
- extinction of light
- decomposition of detrital organic matter
- consumption of electron acceptors, methanogenesis
- nitrification, denitrification
- adsorption, precipitation of phosphate
- dissolution of opal silicate
- oxidation, precipitation, speciation of sulphide
- oxidation, ebullition, volatilization of methane
- reaeration of dissolved oxygen
- net settling of particulate components
- mass transport in the sediment

**Figure 1:**
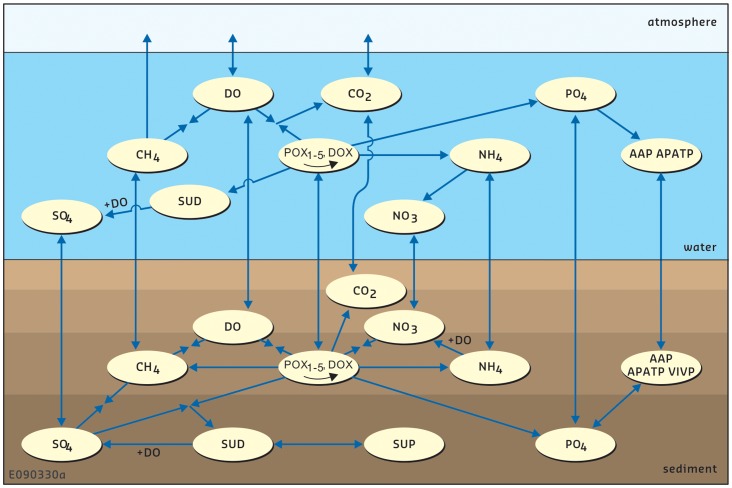
Schematic overview of the state variables and processes included in ECO. Processes for phytoplankton and silicate are not included. Carbon dioxide is not included in the Lake Veluwe model.

Figure 1 provides an overview of the substances and processes. Tables 1, 2, 3, 4 specify the mathematical formulations and the symbols. The reaction term P in the above advection diffusion equation is given for all substances in Equations A.1–16. The formulations for the individual processes and parameters are presented in sections B-G of Tables 1, 2. The process formulations are generic for water column and sediment bed, but may turn out differently in individual grid cells depending on local chemical conditions such as the presence or absence of dissolved oxygen.

**Table 1:** The formulations in ECO (part 1).

A. The processes terms for state variables (term P in equation 1)	
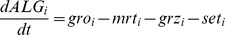	(A.1)
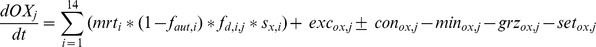	(A.2)
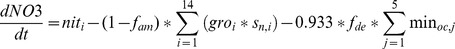	(A.3)
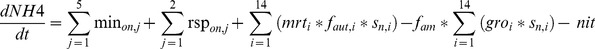	(A.4)
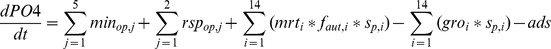	(A.5)
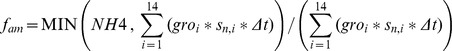	(A.6)
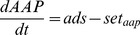	(A.7)
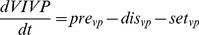	(A.8)
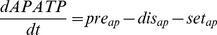	(A.9)
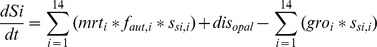	(A.10)
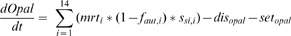	(A.11)
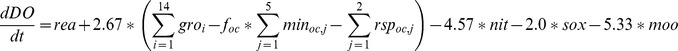	(A.12)
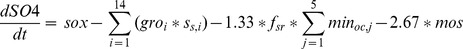	(A.13)
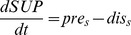	(A.14)
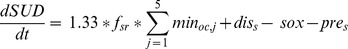	(A.15)
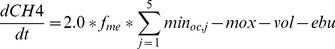	(A.16)
B. Decomposition/conversion of organic matter, consumption of electron acceptors	
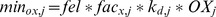	(B.1)
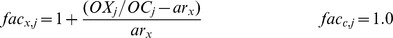	(B.2)
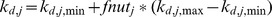	(B.3)
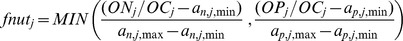	(B.4)
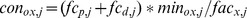	(B.5)
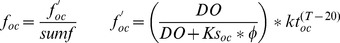	(B.6)
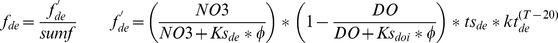	(B.7)
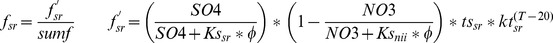	(B.8)
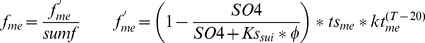	(B.9)
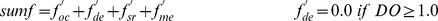	(B.10)
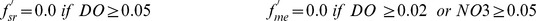	(B.11)
C. Nutrient processes	
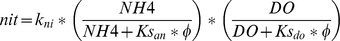	(C.1)
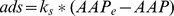	(C.2)
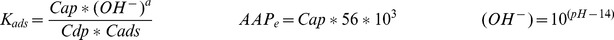	(C.3)

**Table 2:** The formulations in ECO (part 2).

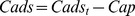	(C.4)
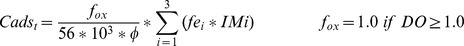	(C.5)
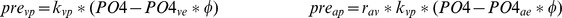	(C.6)
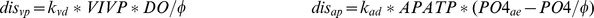	(C.7)
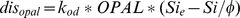	(C.8)
**D. Sulphur and methane processes**	
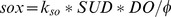	(D.1)
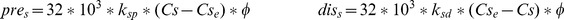	(D.2)
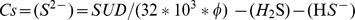	(D.3)
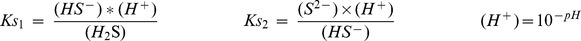	(D.4)
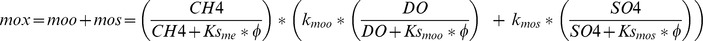	(D.5)
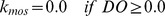	(D.6)
**E. Exchange of gases with the atmosphere**	
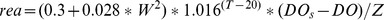	(E.1)
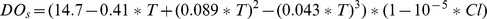	(E.2)
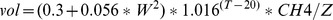	(E.3)
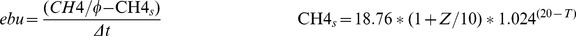	(E.4)
**F. Settling and mass transport in the sediment settling**	
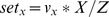	(F.1)
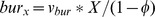	(F.2)
	(F.3)
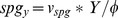	(F.4)
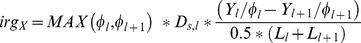	(F.5)
**G. Extinction of light**	
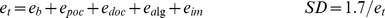	(G.1)
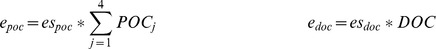	(G.2)
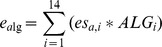	(G.3)
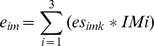	(G.4)

where :

*i* **= **algae types 1–14.

*j* **= **particulate detrital organic matter fractions 1–4, and dissolved fraction 5.

*l* = sediment layer 1–7, overlying water layer 0 (L_0_ = 0.0005 m).

*OX* = particulate (POX_1–4_) or dissolved (DOX) detrital fraction; X is C, N, P or S.

*X* = any particulate substance; ALG*i*, POX, AAP, VIVP, APATP, OPAL, IM1, IM2.

*Y* = any dissolved substance.

**Table 3:** Explanation of the symbols in Tables 1–2, with values of the process coefficients in ECO (part 1).

Symbol	Description	Value	Unit
	*State variables*		
ALG_1_	diatoms energy type	*	g C.m^−3^
ALG_2_	diatoms phosphorus type	*	g C.m^−3^
ALG_3_	green algae energy type	*	g C.m^−3^
ALG_4_	green algae nitrogen type	*	g C.m^−3^
ALG_5_	green algae phosphorus type	*	g C.m^−3^
ALG_6_	*Aphanizomenon* energy type	*	g C.m^−3^
ALG_7_	*Aphanizomenon* nitrogen type	*	g C.m^−3^
ALG_8_	*Aphanizomenon* phosphorus type	*	g C.m^−3^
ALG_9_	*Microcystis* energy type	*	g C.m^−3^
ALG_10_	*Microcystis* nitrogen type	*	g C.m^−3^
ALG_11_	*Microcystis* phosphorus type	*	g C.m^−3^
ALG_12_	*Oscillatoria* energy type	*	g C.m^−3^
ALG_13_	*Oscillatoria* nitrogen type	*	g C.m^−3^
ALG_14_	*Oscillatoria* phosphorus type	*	g C.m^−3^
POC/N/P/S1	fast decomposing particulate organic C/N/P/S	*	g C/N/P/S.m^−3^
POC/N/P/S2	moderately slow decomposing particulate organic C/N/P/S	*	g C/N/P/S.m^−3^
POC/N/P/S3	slow decomposing particulate organic C/N/P/S	*	g C/N/P/S.m^−3^
POC/N/P/S4	very slow decomposing (refractory) particulate organic C/N/P/S	*	g C/N/P/S.m^−3^
DOC/N/P/S	very slow decomposing (refractory) dissolved organic C/N/P/S	*	g C/N/P/S.m^−3^
NO3	nitrate	*	g N.m^−3^
NH4	ammonium	*	g N.m^−3^
PO4	dissolved phosphate	*	g P.m^−3^
AAP	adsorbed phosphate	*	g P.m^−3^
VIVP	vivianite-like phosphate	*	g P.m^−3^
APATP	apatite-like phosphate	*	g P.m^−3^
Si	dissolved silicate	*	g Si.m^−3^
OPAL	opaline silicate	*	g Si.m^−3^
DO	dissolved oxygen	*	g O_2_.m^−3^
SO4	sulphate	*	g S.m^−3^
SUD4	dissolved sulphide	*	g S.m^−3^
SUP	particulate sulphide	*	g S.m^−3^
CH4	methane	*	g C.m^−3^
IM1/2/3	silt in sediment/sand in sediment/silt in water	*	g.m^−3^
Cl	chloride	*	g Cl.m^−3^
	***Fluxes***		
gro	net phytoplankton growth (gross production – respiration)	*	g C.m^−3^.d^−1^
mrt	phytoplankton mortality	*	g C.m^−3^.d^−1^
grz	grazing by zooplankton	*	g X.m^−3^.d^−1^
rsp/exc	respiration/excretion by zooplankton	*	g X.m^−3^.d^−1^
min/con	mineralization/conversion of detrital organic matter	*	g X.m^−3^.d^−1^
upt	uptake of nutrients by phytoplankton	*	g X.m^−3^.d^−1^
nit	nitrification	*	g N.m^−3^.d^−1^
ads	adsorption of phosphate	*	g P.m^−3^.d^−1^
pre/dis	precipitation/dissolution	*	g X.m^−3^.d^−1^
sox	sulphide oxidation	*	g S.m^−3^.d^−1^
mox/moo/mos	methane oxidation/with oxygen/with sulphate	*	g C.m^−3^.d^−1^
rea	reaeration	*	g O_2_.m^−3^.d^−1^
vol/ebu	volatilization/ebullition of methane	*	g C.m^−3^.d^−1^
set	settling	*	g X.m^−3^.d^−1^
bur/tur	burial/bioturbation of particulate substances in the sediment	*	g X.m^−3^.d^−1^
spg/irg	seepage/bioirrigation of dissolved substances in the sediment	*	g X.m^−3^.d^−1^
	***Process coefficients***		
a	reaction constant pH-dependency of phosphate adsorption	0.2	-
a_n,1,min/max_	min/max nitrogen content for decomposition rate POX1	0.075/0.15	g N.g C^−1^
a_n,2,min/max_	min/max nitrogen content for decomposition rate POX2	0.06/0.12	g N.g C^−1^
a_p,1,min/max_	min/max phosphorus content for decomposition POX1	0.0075/0.015	g P.g C^−1^
a_p,2,min/max_	min/max phosphorus content for decomposition POX2	0.006/0.012	g P.g C^−1^
AAP_e_	equilibrium adsorbed phosphate	*	g P.m^−3^
ar_n_	target minimal nitrogen content of refractory detritus	0.07	g N.g C^−1^
ar_p_	target minimal phosphorus content of refractory detritus	0.007	g P.g C^−1^
Cads_(t)_	free (total) adsorbent for phosphate	*	mol.L^−1^
Cap	adsorbed phosphate	*	mol.L^−1^
Cdp	dissolved phosphate	*	mol.L^−1^
CH4_s_	saturation methane concentration	*	g C.m^−3^(w)
Cs_e_	free sulphide concentration at saturation for FeS	0.2 10^−10^	mol.L^−1^
D_p_	dispersion coefficient for particulates (bioturbation)	**	m^2^.d^−1^
D_s_	dispersion coefficient for solutes (bioirrigation, diffusion)	**	m^2^.d^−1^
DO_s_	saturation concentration of dissolved oxygen	*	g O_2_.m^−3^
e_alg_	algae biomass extinction	*	m^−1^
e_b_	background extinction of fresh water	0.08	m^−1^
e_doc_	dissolved organic matter extinction	*	m^−1^
e_im_	inorganic sediment extinction	*	m^−1^
e_poc_	specific extinction of particulate organic detritus	*	m^−1^
e_t_	total extinction	*	m^−1^
es_a,i_	specific extinction of algae type ALG_i_	***	m^2^.g C^−1^
es_doc_	specific extinction of dissolved organic matter	0.3	m^2^.g C^−1^
es_poc_	specific extinction of particulate organic detritus	0.1	m^2^.g C^−1^

* Calculated in ECO,

** temporally and/or spatially varying forcing parameter for ECO,

*** values in Table 5.

**Table 4:** **E**xplanation of the symbols in Tables 1–2, with values of the process coefficients in ECO (part 2).

Symbol	Description	Value	Unit
es_im3_	specific extinction of suspended inorganic matter	0.018	m^2^.g^−1^
f_am_	fraction ammonium in nitrogen uptake by phytoplankton	*	–
f_aut,i_	autolysis fraction of mortality of ALG_i_	0.35	–
f_d,i,1_	fraction allocated to detritus fraction POX1 (i = 1−8/9−14)	0.55/0.62	–
f_d,i,2_	fraction allocated to detritus fraction POX2 (i = 1−8/9−14)	0.45/0.38	–
f_de_	fraction of detritus decomposed with denitrification	*	–
f_me_	fraction of detritus decomposed with methanogenesis	*	–
f_oc_	fraction of detritus decomposed with oxygen consumption	*	–
f_ox_	fraction of oxidized reactive iron at reducing conditions	****	–
f_sr_	fraction of detritus decomposed with sulphate reduction	*	–
fac_x,j_	factor for nutrient stripping (x = N or P) for OX_j_	*	–
fc_d,j_	fraction conversion into diss. detritus (POX2/3→DOX)	0.17	–
fc_p,j_	fraction conversion of part. detritus (1→2/2→3/3→4)	0.3/0.2/0.2	–
fe	fraction of reactive iron in bottom sediment (IM1/IM2)	****/0.0	–
fe_i_	fraction of reactive iron in susp. sediment (IM1/IM2/IM3)	0/0/0.05	–
fel	factor for dominant electron acceptor, relative to oxygen	1.0	–
fnut	function for relative nutrient availability for bacteria	*	–
I_z_	light (solar radiation) as a function depth	**	W.m^−2^
k_ad_	apatite dissolution rate	0.0025	(g P.m^−3^)^−1^.d^−1^
k_d,j_	decomposition rate for organic matter OX_j_	*	d^−1^
k_d,1,min/max_	min/max mineralization rate for POX1 (values at 20°C)	0.15/0.2	d^−1^
k_d,2,min/max_	min/max mineralization rate for POX2 (value at 20°C)	0.025	d^−1^
k_d,3,min/max_	min/max mineralization rate for POX3 (value at 20°C)	0.0012	d^−1^
k_d,4,min/max_	min/max mineralization rate for POX4 (value at 20°C)	0.000035	d^−1^
k_d,5_	mineralization rate for DOX (value at 20°C)	0.002	d^−1^
k_moo_	methane oxidation rate with oxygen (value at 20°C)	0.1	g C.m^−3^.d^−1^
k_mos_	methane oxidation rate with sulphate (value at 20°C)	0.05	g C.m^−3^.d^−1^
k_ni_	nitrification rate in water/sediment at 20°C)	0.2/25.0	g N.m^−3^.d^−1^
k_od_	opal dissolution rate	0.00005	(g Si.m^−3^)^−1^.d^−1^
k_s_	adsorption/desorption rate for phosphate (value at 20°C)	10.0	d^−1^
k_so_	sulphide oxidation rate	10.0	(g O_2_.m^−3^)^−1^.d^−1^
k_sd_	sulphide dissolution rate	2.0 10^6^	d^−1^
ks_p_	sulphide precipitation rate	10^6^	d^−1^
kv_d_	vivianite dissolution rate	0.05	(g O_2_.m^−3^)^−1^.d^−1^
kv_p_	vivianite precipitation rate	0.6	d^−1^
km_i_	mortality rate of ALG_i_ (value at 0°C)	***	d^−1^
kp_i_	maximal net growth rate of ALG_i_ (value at 0°C)	***	d^−1^
kr_i_	maintenance respiration rate of ALG_i_ (value at 0°C)	***	d^−1^
kt	temperature coefficients	****	–
kt_m,i_	temperature coefficient of mortality of ALG_i_	***	–
kt_p,i_	temperature coefficient of growth of ALG_i_	***	–
kt_r,i_	temperature coefficient of maintenance respiration of ALG_i_	1.072	–
K_ads_	phosphate adsorption constant	2.2 10^3^	(mol.L^−1^)^a−1^
Ks_an_	half saturation constant for ammonium in nitrification	0.4	g N.m^−3^
Ks_do_	half saturation constant for oxygen in nitrification	1.0	g O_2_.m^−3^
Ks_de_	half saturation constant for denitrification	0.25	g N.m^−3^
Ks_oc_	half saturation constant for oxygen consumption	1.0	g O_2_.m^−3^
Ks_sr_	half saturation constant for sulphate reduction	2.0	g S.m^−3^
Ks_doi_	half saturation constant for denitrification inhibition	1.0	g O_2_.m^−3^
Ks_nii_	half saturation constant for sulphate reduction inhibition	0.02	g N.m^−3^
Ks_sui_	half saturation constant for methanogenesis inhibition	1.0	g S.m^−3^
Ks_me_	half saturation constant for methane in methane oxidation	0.5	g C.m^−3^
Ks_moo_	half saturation constant for oxygen in methane oxidation	1.0	g O_2_.m^−3^
Ks_mos_	half saturation constant for sulphate in methane oxidation	1.0	g S.m^−3^
Ks_1/2_	acidity constants for sulphide	10^−7^/10^−14^	L.mol^−1^
L	layer thickness	**	m
pH	acidity in water/sediment	**/7.0	–
(H^+^)	hydronium ion concentration	*	mol.L^−1^
(OH^−^)	hydroxyl ion concentration	*	mol.L^−1^
PO4_ae_	equilibrium dissolved phosphate concentration for apatite	0.15	g P.m^−3^(w)
PO4_ve_	equilibrium dissolved phosphate concentration for vivianite	0.15	g P.m^−3^(w)
r_av_	ratio of apatite and vivianite precipitation	2.0	–
s_x/chl/n/p/si/s,i_	stoichiometric constant for X/Chl/N/P/Si/S in ALG_i_	***	g X.g C^−1^
SD	secchi depth	*	m
Si_e_	equilibrium dissolved silicate concentration for opal	10.0	g Si.m^−3^(w)
(S^2−^)	free sulphide ion concentration	*	mol.L^−1^
(HS^−^)	hydrogen sulphide ion concentration	*	mol.L^−1^
(H2S)	dissolved hydrogen sulphide concentration	*	mol.L^−1^
T	water temperature	**	?C
ts	scaling factors for temperature function ( = 1.0 if T>2 ^o^C)	1.25	–
v	settling velocity of particulate matter (POC, AAP, OPAL, IM1)	****	m.d^−1^
v_a,i_	settling velocity of ALG_i_	0.0	m.d^−1^
v_bur_	burial velocity of particulate substances	*	m.d^−1^
v_spg_	seepage velocity	**	m.d^−1^
W	wind velocity	**	m.s^−1^
Z	water depth	*	m
?t	computational time step	5	minutes
φ	porosity	****	m^3^.m^−3^

* Calculated in ECO,

** temporally and/or spatially varying forcing parameter for ECO,

*** values in Table 5,

**** values in the text.

Compared to DBS [26], the process formulations for organic matter decomposition, electron acceptor consumption, nitrification, phosphate adsorption. phosphate mineral formation and opal dissolution have been improved, and the sulphur and methane processes have been added.

### Phytoplankton Processes

Phytoplankton is simulated with BLOOM as was done previously with DBS [26]. This model and the calibration of phytoplankton properties were reported in full detail by Los [34], its concepts and main formulations also by Los & Wijsman [35] and Blauw et al. [24]. Therefore, only the overall term P for algae biomass is included in Table 1 (Eq. A.1). BLOOM allows for the modeling of the competition and adaptation of phytoplankton species to limiting nutrients and light regime. For the simulation of species competition in freshwater two algae groups (diatoms, green algae) and three cyanobacteria species (*Microcystis*, *Aphanizomenon* and *Oscillatoria/Planktothrix*) have been defined in ECO. A species (group) has three phenotypes to account for adaptation to changing environmental conditions:

- the energy type, with relatively high growth rate, low mortality rate and high N/C and P/C ratio;
- the nitrogen type, typically with lower internal N/C ratio, lower maximum growth rate, higher mortality rate, higher settling velocity and higher chlorophyll-a content; and
- the phosphorus type, typically with lower internal P/C ratio, lower maximum growth rate, higher mortality rate, lower settling velocity and lower chlorophyll-a content.

The biomasses of the phenotypes are modelled as 14 separate variables (ALG_i_) with different nutrient and chlorophyll-a contents, extinction coefficient, growth rate, respiration rate, mortality rate and settling velocity (Table 5; [34]). Additional coefficients concern the fractions for autolysis of nutrients and production of detritus (Tables 3, 4; [34]). When ambient conditions change, a phenotype can be instantaneously converted into another phenotype of the same species, representing rapid adaptation of algal cells. In BLOOM the species compete within the constraints for available nutrients (N, P, Si), available light (energy), the maximum growth rate and the maximum mortality rate (both temperature functions). Linear programming is used as an optimization technique to determine the species composition that is best adapted to prevailing environmental conditions. It can be shown mathematically that the principle by which each phytoplankton type maximizes its own benefit effectively means that the total net production of the phytoplankton community is maximized [34]. Process fluxes are calculated on the basis of daily averaged meteorological forcing. Therefore, the simulation time step for the BLOOM phytoplankton processes is 24 hours. Phytoplankton settled in the sediment dies according to BLOOM’s mortality rate. Grazing by zooplankton is taken into account by means of a grazing rate that operates on algae biomass (ALG_i_) as well as detritus (POC/N/P). This rate is proportional to imposed zooplankton biomass as described by [24].

**Table 5:** Parameter values in the algal module (BLOOM) of ECO for freshwater algae (Los, 2009).

ALG_i_	es_a,i_	s_n,i_	s_p,i_	s_si,i_	s_chl,i_	kp_i_	kt_p,i_	km_i_	kt_m,i_	kr_i_
	[m^2^.g.C^−1^]	[g.g^−1^]	[g.g^−1^]	[g.g^−1^]	[g.g^−1^]	[d^−1^]	[−]	[d^−1^]	[−]	[d^−1^]
Diat-E	0.2700	0.210	0.0180	0.6600	0.040	0.350	1.060	0.035	1.080	0.031
Diat-P	0.1875	0.188	0.0113	0.5500	0.025	0.350	1.054	0.045	1.085	0.031
Green-E	0.2250	0.275	0.0238	0.0018	0.033	0.068	0.000	0.035	1.080	0.031
Green-N	0.1875	0.175	0.0155	0.0018	0.025	0.068	3.000	0.045	1.085	0.031
Green-P	0.1875	0.200	0.0129	0.0018	0.025	0.068	3.000	0.045	1.085	0.031
Aphan-E	0.4500	0.220	0.0125	0.0018	0.033	0.190	1.083	0.035	1.080	0.012
Aphan-N	0.4000	0.125	0.0125	0.0018	0.025	0.150	1.095	0.045	1.085	0.012
Aphan-P	0.4000	0.170	0.0088	0.0018	0.025	0.150	1.095	0.045	1.085	0.012
Micro-E	0.4000	0.225	0.0300	0.0018	0.025	0.056	3.000	0.035	1.080	0.012
Micro-N	0.2875	0.113	0.0275	0.0018	0.017	0.048	5.000	0.045	1.085	0.012
Micro-P	0.2875	0.175	0.0225	0.0018	0.017	0.048	5.000	0.045	1.085	0.012
Oscil-E	0.4000	0.225	0.0188	0.0018	0.033	0.045	0.000	0.035	1.080	0.012
Oscil-N	0.2875	0.125	0.0138	0.0018	0.020	0.034	0.000	0.045	1.085	0.012
Oscil-P	0.2875	0.150	0.0113	0.0018	0.020	0.034	0.000	0.045	1.085	0.012

Settling velocities v_a,i_ = 0.0 m.d^−1^, temperature coefficients kt_r,i_ = 1.072, stoichiometric constants s_s,i_ = 0.0175 g S.g C^−1^.

### Extinction of Light

Light is simulated as photosynthetically active radiation (PAR) [24], [34]. Extinction is modelled as an exponential decrease of light intensity with depth according to the Lambert-Beer formula. The total extinction coefficient is calculated as the sum of the extinction by inorganic suspended matter (IM3 in the Lake Veluwe model), particulate organic matter (POC1–4), dissolved organic matter (DOC), phytoplankton biomass (ALG_1–14_) and water (Eqs. G1–4 in Table 2). Each substance has a specific extinction coefficient. As opposed to other eutrophication models the extinction due to DOC is modeled explicitly.

### Decomposition of Detrital Organic Matter

The decomposition of detrital organic matter is innovatively formulated as the mineralization and conversion of five fractions, allowing for the simulation of the entire organic matter cycle in surface water and bed sediment. POC1 is the fast decomposing detritus fraction, POC2 the moderately slow decomposing fraction, POC3 the slow decomposing fraction and POC4 the very slow decomposing (refractory) fraction. POC1 and POC2 are the dominant fractions in the water column, whereas POC3 and POC4 are the dominant fractions in the sediment. DOC represents very slow decomposing (refractory) dissolved organic matter being produced in both water column and sediment. A mineralization flux may have proportional conversion fluxes, which represent the gradual turnover of detritus into residual refractory organic matter due to enzymatic stripping and polymerization. In the Lake Veluwe model POC/N/P/S1 is converted into POC/N/P/S2, POC/N/P/S2 into POC/N/P/S3 and DOC/N/P/S, and POC/N/P3 into POC/N/P4 and DOC/N/P/S. This decomposition and conversion scheme is an extension of the multi-G model [32] applied in various forms in many models [2], [17], [20], [30], [36]. Raick et al. [37], Lancelot et al. [8] and Dale et al. [38] describe different versions of an alternative approach based on the hydrolysis of particulate and dissolved polymeric detritus fractions into monomeric dissolved substances that are taken up and respired by bacteria. This delivers essentially similar results as the approach in ECO, because both concepts have the same rate limiting first decomposition step.

The mineralization rate in ECO conforms to first-order kinetics [25], [32], [39]. The rate is dependent on the availability of the nutrients (N, P) needed by detritus decomposing bacteria [24], and on the dominant electron acceptor [19], [40] (Eqs. B.1, B.3, B.4). The latter dependency was ignored in the Lake Veluwe model. Mineralization in ECO implies the release of ammonium (NH4), phosphate (PO4) and sulphide (SUD), the consumption of electron-acceptors (DO, NO3, SO4) and/or methanogenesis (CH4). Preferential stripping of nutrients from detritus is taken into account by means of an acceleration factor for organic nutrients (Eq. B.2), which is proportional to the difference of actual and objective detritus nutrient contents [24], [26]. The objective contents are the low nutrient contents of humic and fulvic matter, the residual products of the decomposition process. The conversion of detritus is proportional to mineralization with ratios specific for each of the detritus fractions (Eq. B.5).

### Consumption of Electron Acceptors and Methanogenesis

The dominant electron acceptors consumed for the oxidation-mineralization of organic matter are oxygen (DO), nitrate (NO3), sulphate (SO4) and carbon monoxide. In the model, carbon monoxide and hydrogen arising from the fermentation of organic matter at chemically reducing conditions are implicit in the decomposition product methane. The electron acceptors iron(III) and manganese(IV) are assumed to be implicit in the sulphur redox cycle. This can be justified from the poor solubility of their oxy-hydroxides, and the coupling of the metal and sulphur cycles as described by Wang & Van Cappellen [14] and Wijsman et al. [18]. As ruled by redox potential dependent energy yields, different groups of bacteria species consume the electron acceptors in a specific sequence [12], [13], [41]. When dissolved oxygen is not available, bacteria resort to nitrate for the decomposition of organic matter. If both oxygen and nitrate are depleted, sulphate is used by other bacteria. At the depletion of all three substances methanogenic bacteria convert organic matter into approximately equal amounts of methane and carbon dioxide.

The consumption of an electron acceptor is limited by availability and inhibited by the presence of electron acceptors that yield more energy. However, because both sediment and water compartments are not homogenously mixed in the real world, the electron acceptors may be depleted in parts of the compartments, implying that they can be consumed simultaneously in each model compartment. The sequential but spatially overlapping consumption of electron acceptors is reproduced in ECO with Michaelis-Menten kinetics for limitation and inhibition [12], [13], [17]–[19], [20], [22], [30], which is a step forward with regard to the formulation of sediment diagenesis in sediment module SWITCH [25] of DBS and in other eutrophication models. Simplified limitation and inhibition functions were described by Aguilera et al. [42] and applied by Brigolin et al. [2]. Dale et al. [38] presented an alternative approach based on double Michaelis-Menten kinetics for electron donors and acceptors and Gibbs free energy yields. In ECO denitrification is inhibited by oxygen, sulphate reduction by nitrate and oxygen, and methanogenesis by sulphate, nitrate and oxygen (Eqs. B.6–11). The Michaelis-Menten factors are scaled to make the sum of the factors equal 1.0. The total consumption rate of electron acceptors is proportional to the mineralization rate of organic matter (Eqs. A3, A12, A23, A16).

### Nitrification

Ammonium (NH4) is oxidized to nitrate (NO3) by means of stepwise nitrification, the oxidation of nitrite being the last and rate limiting step [13], [18], [19]. Because nitrifying bacteria need readily available organic substrates, nitrification proceeds most rapidly in the oxic top sediment layer. Nitrification is often described with first-order kinetics with regard to ammonium [25], [26], [43] or to both ammonium and oxygen [2], [17], [30]. In ECO nitrification is formulated according to Michaelis-Menten kinetics with respect to ammonium and dissolved oxygen (Eq. C.1).

### Phosphate Processes

The adsorption of phosphate (PO4) to iron(III) oxyhydroxides and other inorganic components of sediment is a pH dependent equilibrium process [44]. Desorption occurs when pH increases. The adsorption capacity of sediment depends on the presence of oxygen. At anoxic conditions the capacity is far smaller, mainly due to the reduction of iron(III) oxyhydroxides, but some adsorption capacity is maintained in sediment [9], [23], [28], [45], [46]. The adsorption of phosphate can be described in various ways with linear or Langmuir equilibrium sorption [25], [26], [28], [47]. In ECO, phosphate adsorption is formulated according to a pH dependent Langmuir model according to Eqs. C.2–5 [44]. The pH is a forcing function. Innovatively, the adsorption capacity is deduced from the reactive iron content of the sediment corrected for the oxidized iron(III) fraction. The correction factor is equal to 1.0 when the oxygen concentration is above a critical value, but smaller at lower oxygen concentrations. The sorption flux is calculated from the difference of the actual and equilibrium adsorbed phosphate concentrations multiplied with a sorption rate. The equilibrium adsorbed phosphate concentration follows from Equation C.3 (Table 1).

In the sediment vivianite (iron(II) phosphate) or apatite-(calcium phosphate)-like minerals may precipitate at the supersaturation of pore water [48]. Boers & de Bles [49] studied the possible precipitation of vivianite in Lake Loosdrecht (peat lake), but did not find conclusive evidence. However, Dittrich et al. [19] included vivianite in their sediment diagenesis model for Lake Zug. The formation of Ca-P minerals in marine sediment has been stipulated by De Jonge [50], Slomp et al. [23], Slomp & Van Cappellen [51] and Anschutz et al. [45]. However, the coprecipitation of phosphate with calcite is not always found for marine sediment [28]. Unlike vivianite that is only stable under anoxic conditions, the stability of calcium phosphates is not affected by the redox potential. Therefore, the precipitation of apatite-like minerals may lead to the more or less permanent storage of phosphate in sediment [23]. The mechanisms and controlling factors of P-mineral formation are not well understood [46]. Generally, the precipitation and dissolution rates are modeled proportional to the difference of an ion activity product and the solubility product, whereas the dissolution rate is also proportional to the concentration of the mineral [12]–[14], [18], [19]. In ECO the precipitation of phosphate is formulated in a simplified way with first-order kinetics [23], [25], the difference between the actual and equilibrium dissolved phosphate concentrations being the driving force (Eq. C.6). This is justified by the assumption that the pH in the sediment and the pore water concentration profiles of iron(II) and calcium are rather constant, although iron(II) varies in the oxidizing-reducing transition zone. Unlike other eutrophication models ECO distinguishes between vivianite-P and apatite-P. In ECO, the precipitation of vivianite takes place only when the average dissolved oxygen concentration is below a critically low value (0.25 mg O_2_/L). The dissolution rate of the apatite-like mineral depends on the extent of undersaturation as well as the concentration of the mineral (Eq. C.7). The dissolution of the vivianite-like mineral is proportional to the concentrations of the mineral and dissolved oxygen.

### Dissolution of Silicate

In many models the dissolution of opal silicate (OPAL) is dealt with as first-order degradation [8], [24], [37]. However, this is a chemical-physical process proceeding in an undersaturated solution [11], [52], [53]. In ECO, OPAL is slowly converted into dissolved silicate (Si) proportional to the concentration of OPAL and the difference between the saturation and actual dissolved silicate concentrations (Eq. C.8).

### Sulphide Processes

The oxidation of sulphide is established by fast chemical reaction and slow microbial conversion [14], [18]. The oxidation of dissolved sulphide is modeled as a first-order process with regard to both total dissolved sulphide (SUD) and dissolved oxygen (DO) [17], [19], [30]. At reducing conditions sulphide may precipitate as amorphous iron(II) sulphide, which is thermodynamically unstable and dissolves at oxidizing conditions. The precipitation and dissolution rates are usually formulated on the basis of the difference of the ion activity and solubility products [13], [14], [19], [44], but also with first order kinetics with respect to dissolved Fe^2+^ and H_2_S [15], [30]. In ECO however, the precipitation of sulphide (SUD) and the dissolution of particulate sulphide (SUP) is described with first-order kinetics (Eqs. D.2–4). The difference between the actual and equilibrium free sulphide concentrations is the driving force. It is assumed that sulphide forming metals like iron(II) are abundantly present in the sediment. The free dissolved sulphide concentration (S^2−^) is calculated from the total dissolved sulphide concentration and the pH. The chemical equilibrium reactions and pertinent equilibrium constants were derived from Stumm & Morgan [44].

### Methane Oxidation

Bacteria oxidize methane with dissolved oxygen (DO) or sulphate (SO4). Often first-order kinetics with regard to both methane and oxygen are applied [14], [17], [18], [30]. In ECO, methane oxidation is modeled according to Michaelis-Menten kinetics with regard to both methane and the oxidizing agent (Eqs. D.5–6), whereas the oxidation with dissolved oxygen excludes the oxidation with sulphate [13].

### Exchange of Gases with the Atmosphere

The formulation of the diffusion flux of oxygen (DO: reaeration) and methane (CH4: volatilization) across the water surface is based on the double film concept. The flux is the temperature dependent mass transfer coefficient in water multiplied with the difference of the saturation and actual concentrations, the concept of which was described by Wanninkhof [54]. In the Lake Veluwe model, the wind speed dependent formulation for the transfer coefficient according to Banks & Herrera [55] is used (Eqs. E.1–3). The saturation concentration of oxygen is calculated according to the temperature and chlorinity dependent formulation described by Weiss [56]. Additionally, methane may escape to the atmosphere via gas bubbles, which are formed in the sediment at the supersaturation of dissolved methane. Similar to the approach of Canavan et al. [20], it is assumed that all methane formed at supersaturation is removed instantly (Eq. E.4). The saturation concentration depends on pressure (water depth) and follows from the formulation described by Di Toro [13].

### Settling and Mass Transport in the Sediment

The settling flux of a particulate component is proportional to its concentration and settling velocity (Eq. F.1). In the Lake Veluwe model we consider time average net settling (no resuspension), and the settling velocities for algae are equal to 0.0.

Mass transport in the sediment results from advection and dispersion [11], [13]. The dispersion and advection of dissolved substances across the sediment-water interface imply return fluxes of nutrients to the water column and a sediment oxygen demand. Advection arises from upward or downward seepage of solutes, and from settling and resuspension of particulates. Settling leads to burial, resuspension to upward transport. The latter requires that sediment and water quality at the lower boundary of the sediment are defined. In the Lake Veluwe model burial results from settling at constant porosity and constant sediment layer thickness. Burial and seepage fluxes follow from Equations F.2–3. Dispersion is caused by seasonally varying activity of benthic organisms resulting in bioturbation of particulates and bio-irrigation of solutes. Solutes are also transported due to flow induced micro turbulence and molecular diffusion [57]. In ECO, dispersion is calculated as diffusive transport according to Fick’s second law [11] (Eqs. F.4–5). The dispersion coefficient of solutes is the sum of the bio-irrigation coefficient, the flow induced dispersion coefficient and the molecular diffusion coefficient. Except for the latter which is corrected for tortuosity, all coefficients decrease exponentially over depth in the Lake Veluwe model.

### Process Coefficients and Stochiometric Constants

Tables 3, 4 present the values of the process coefficients in the ECO model for Lake Veluwe. All rate constants are temperature dependent according to:
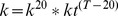
where

k^20^ = rate constant at 20 ^o^C (d^−1^ or g.m^−3^.d^−1^)

kt = temperature coefficient (−)

T = temperature (°C)

The temperature constants for the decomposition of organic matter and other microbial processes are 1.047 and 1.07, respectively. Apart from the dissolution of opal silicate (kt = 1.07), the temperature constants of precipitation and dissolution processes are equal to 1.0. Stochiometric constants for the various redox processes are specified in Equations A.3, A.12–14 and A.16. The stochiometric constants s_x,i_ for algae biomass in Equations A.2–6 and A10–11 are given in Table 5.

## The Calibration of ECO

### The Lake Veluwe case

**Figure 2:**
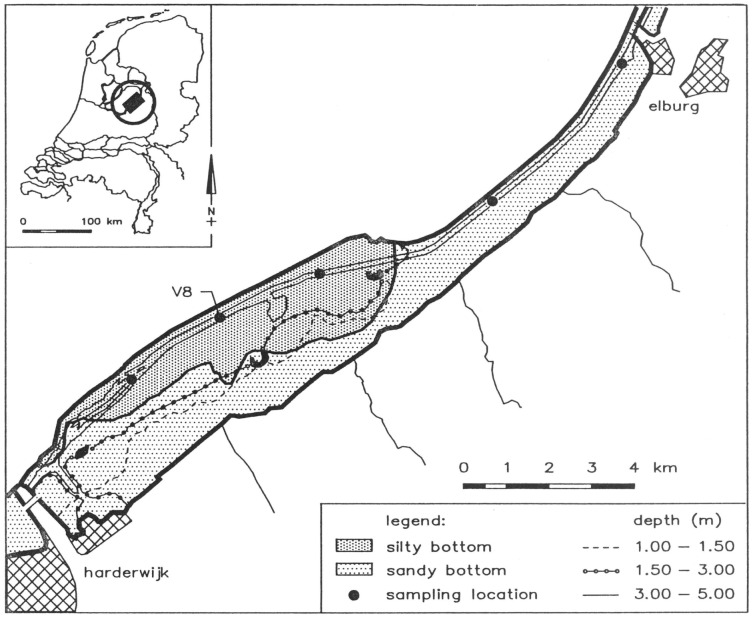
Lake Veluwe and its surroundings.

Lake Veluwe (The Netherlands) is a shallow lake which was formed when the Flevoland polder was created in 1956 (Figure 2; [58]). The lake has a total surface area of 32.3 km^2^. 60% of the lake has a sandy sediment with approximately 1.5% calcite at an average depth of 0.75 m. The other 40% has a silty sediment with approximately 8% calcite at an average depth of 2.5 m [59]. Bordering higher land, the shallow part has seepage. The deeper part has infiltration due to the low Flevoland polder in the northwest [60]. In the second half of the 1960s the water quality of the lake deteriorated, because of increased nutrient loading. As of 1970, this resulted in high chlorophyll-a concentrations and an almost permanent bloom of cyanobacteria, predominantly *Oscillatoria agardhii*. From February 1979 onwards, external phosphorus loading was reduced from 3 to 1–1.5 g P.m^−2^.yr^−1^ by means of phosphorus removal at the sewage treatment plant discharging its effluent to the lake. Additionally, the lake was flushed in winter with water from the Flevoland polder (poor in algae and phosphorus, but rich in calcium and nitrate), which reduced the average winter half year residence time of dissolved compounds from 0.35 to 0.15 year. In the second half of the 1980s, the lake was flushed in summer as well, reducing the average summer half year residence time of dissolved compounds from 0.5 to 0.25 year. Consequently, total phosphorus and chlorophyll-a concentrations decreased as of 1980, reaching strongly reduced levels in the second half of the 1980s. Whereas diatoms and green algae became dominant over cyanobacteria, submerged macrophytes began to re-appear as of 1985 [60], [61].

### Model Input, Schematization and Forcing

The DBS model of Lake Veluwe had one water compartment and 4 sediment layers [25], [26]. For the ECO model, Lake Veluwe was divided into a “shallow” compartment and a “deep” compartment, that have the above mentioned depths and areas, and different net settling velocities and sediment compositions. Each compartment has 10 sediment layers, the thicknesses of which increase with sediment depth: 4×0.1 cm (1–4), 0.2 cm (5), 0.4 cm (6), 1.0 cm (7), 2.0 cm (8), 6.0 cm (9), 10.0 cm (10). Layer 10 serves as a buffer layer. Sediment porosity and the initial composition of the sediment with regard to organic matter and inorganic P were based on measurement data for 1979–1981 [59], [60]. The porosities for the sandy sediment in the shallow compartment and the silty sediment in the deep compartment are 0.4 and 0.7, respectively. Starting from measured organic matter and total phosphorus contents, the initial sediment composition was adjusted until the model showed a stable composition. The initial composition of the water column followed from the fortnightly routine monitoring data from Rijkswaterstaat, that were also used for the calibration of the model. Inflows and outflows of water were derived from ten days water balances for 1976–1992 based on constant water level (unpublished data from Rijkswaterstaat). The water balance includes inflows from adjacent Lake Wolderwijd, the Flevoland polder, several brooks, the Harderwijk sewage treatment plant, seepage and precipitation. Outflows concern flow to adjacent Lake Drontermeer, infiltration and evaporation. The two compartments exchange water and substances by means of a high dispersion coefficient that warrants very small concentration differences between the two water compartments. Loads of chloride, phosphorus, nitrogen, dissolved silicate and organic matter for each of the inflows are based on observed concentrations (unpublished data from the district water boards Vallei en Veluwe and Zuiderzeeland, made available by Rijkswaterstaat). The sulphate loads on the model were determined proportional to those of chloride. The model is forced with weekly data for solar radiation (PAR; W.m^−2^) and wind speed (m.s^−1^) (data from Royal Netherlands Meteorological Institute), and fortnightly data for water temperature (^o^C), suspended inorganic sediment (IM3; g.m^−3^) and pH (monitoring data from Rijkswaterstaat).

All model input data is available on the OpenEarth model repository. OpenEarth is a free and open source initiative to deal with Data, Models and Tools in water related science & engineering projects [62]. To access the data one needs to sign up on the OpenEarth internet site: http://publicwiki.deltares.nl/display/OET/OpenEarth/. The internet address to the model data is: https://svn.oss.deltares.nl/repos/openearthmodels/trunk/deltares/lake_veluwe_eco/.

### Calibration Procedure

Ethics statement: N/A as to human research, clinical trials, animal research, cell line research, and observational and field studies, because these have not been carried out for this study. No permission for field experiments was required because for this study no field experiments have been carried out. The water quality and water balance data used were collected within the framework of the regular monitoring program of Rijkswaterstaat, who is the management authority for national water systems in the Netherlands such as Lake Veluwe.

Many of ECO’s process coefficients were calibrated for the Lake Veluwe case, whereas stochiometric ratios were fixed at values derived from literature. Initial values were taken from calibrated previous versions of ECO, GEM [24] and DELWAQ-BLOOM-SWITCH [25], [26], or from other reported models. The calibration was done by adjusting the values of process coefficients and by adopting or rejecting new values depending on the improvement or deterioration of model performance, which was assessed by graphical comparison of predicted and observed water and sediment quality parameters. Additional criteria were used for individual coefficients as described below for the resulting coefficients. The calibration was carried out in a step-wise and iterative manner, process by process, going from processes affecting independent state variables to processes affecting dependent state variables, starting from the coefficients for which the model appeared most sensitive. Given the many interactions of substances and processes in the model, this procedure was repeated until no further improvement was observed.

### Resulting Process Coefficients (Tables 3, 4)

In general the individual process coefficients of a calibrated water quality model should have values that are in line with values reported for other similar models. This holds in particular for generic multi coefficient models such as ECO. In view of the generic nature of ECO, the process coefficient values resulting from the calibration are compared with ranges of values reported for other models, both freshwater and saline water models because of the relative scarcity of data for freshwater models.

For a comparison of ECO’s calibrated decomposition and conversion rates of organic detritus (Eqs. B.1–5) with rates reported for other models, values for decomposition in the water column were allocated to k_d,1_ and k_d,2_, and values for decomposition in the sediment to k_d,3_ and k_d,4_. If needed rates were re-calculated for the reference temperature 20 ^o^C (kt = 1.047). For fresh water systems k_d,1_ is between 0.2–0.1 d^−1^, k_d,2_ between 0.04–0.016 d^−1^, k_d,3_ and k_d,4_ between 0.001–0.000026 d^−1^ [19], [20], [28]. For marine water systems k_d,1_ varies between 0.18–0.045 d^−1^, k_d,2_ between 0.075–0.00087 d^−1^, k_d,3_ and k_d,4_ between 0.003–0.0000022 d^−1^ [1], [2], [4], [17], [22], [23], [29], [30], [36], [38], [63]. ECO’s rates are within reported ranges. ECO’s values for target nutrient carbon ratios in refractory detritus ar_n_ and ar_p_ are in line with values reported elsewhere [19], [46], [51].

The coefficients for the consumption of electron acceptors and methanogenesis in ECO (Eqs. B.6–12) were adjusted to obtain vertical concentration profiles in the sediment, that reflect partial overlapping of oxygen consumption, denitrification, sulphate reduction and methanogenesis. ECO’s values for the limitation and inhibition half saturation constants agree with the ranges found in literature for both marine and fresh water systems: Ks_oc_ = 0.05–0.64 mg O_2._L^−1^, Ks_de_ = 0.056–0.42 mg N.L^−1^, Ks_sr_ = 0.0512–32.0 mg S. L^−1^ [1], [2], [17], [19], [20], [22], [29], [30], [38], [63].

We calibrated the rates of nitrification (Eq. C.1) to reproduce observed nitrate and ammonium concentrations and to obtain an appropriate denitrification flux in the sediment. ECO’s nitrification rate for the sediment is within the ranges found in the literature, whereas its value for the water column is somewhat higher. These ranges are for marine water k_ni_ = 0.014–0.07 g N.m^−3^.d^−1^, for marine sediment 2.56–20.0 g N.m^−3^.d^−1^ [1], [2], [4], [8], [17], [22], [24], [29], [30], [36], [37], [63], for fresh water k_ni_ = 0.01 g N.m^−3^.d^−1^, for fresh sediment 9.79–22.4 g N.m^−3^.d^−1^ [6], [19], [20]. To allow comparison with first order, second order and single Michaelis-Menten rates we re-calculated these rates on the basis of temperature (see above; kt = 1.07) and representative Lake Veluwe concentrations of ammonium (1.0 mg N. L^−1^ in sediment and water) and oxygen (4 mg O_2_.L^−1^ in sediment). The ranges of half saturation concentrations for nitrification found in literature are: for marine water Ks_an_ = 0.07–0.28 mg N.L^−1^ and Ks_do_ = 0.16–0.032 mg O_2_.L^−1^ [1], [8], [22], [29], [36], [63] for fresh water Ks_an_ = 0.7 mg N.L^−1^ and Ks_do_ = 0.64 mg O_2_.L^−1^ [19]. ECO’s values for these coefficients are within the overall range (Ks_an_) or slightly higher (Ks_do_).

We found few data in the literature for the coefficients of phosphate processes (Eqs. C.2–7). ECO’s adsorption coefficients were calibrated to reproduce summer dissolved P concentrations in the water column. The adsorption capacity of the sediment must be large to store enough adsorbed phosphate in the top layer to support the high return fluxes in the summers of 1976–1980. The fraction reactive iron fe_i_ in the suspended sediment and the bed sediment was derived from measurement data [59]. The bulk of the sediment has a reactive iron fraction of 0.025 g Fe.g IM1^−1^, the top 4 mm a fraction of 0.075 g Fe.g IM1^−1^ assuming accumulation due to diagenetic processes: Iron is reduced below the top layer and precipitates in the top layer. The fraction of oxidized reactive iron at reducing conditions f_ox_ is 0.2 for the top 4 mm and 0.1 in the deeper sediment. Anschutz et al. [45] found for lagoon sediment, that due to iron reduction the reactive iron(III) concentration decreases to approximately 50% at a depth of 5 cm and to approximately 25% at a depth of 10 cm. ECO’s lower values of f_ox_ for Lake Veluwe sediment are justified by more intensive redox processes due to a much higher organic matter load.

The precipitation of vivianite and apatite-like phosphate minerals is dependent on local chemical conditions. Dittrich et al. [19] determined that the vivianite precipitation rate in a fresh sediment was given by 0.0096 (IAP/SOL-1) g.m^−3^.d^−1^. This can be converted to k_vp_ = 0.8 d^−1^ for PO4 = 2×PO4_ve_, which differs only slightly from ECO’s calibrated value. Slomp et al. [23] present a much lower Ca-P precipitation rate k_vp_ = 0.001 d^−1^ for North Atlantic platform sediment, and an equilibrium concentration PO4_ae_ = 0.11 mg P.L^−1^. Anschutz et al. [45] reported dissolved P concentrations in marine pore water between 0.05 and 0.25 mg P.L^−1^ at the precipitation of apatite. From the data presented by Mort el al. [46] it can be inferred that PO4_ae_ for authigenic Ca-P minerals in the pore water of Baltic Sea sediment could be as high as 4.5 mg P.L^−1^. We selected the equilibrium concentrations PO4_ve_ and PO4_ae_ applied in ECO in line with concentrations observed for pore water in Lake Veluwe sediment [25]. These agree well with the reported ranges. We adjusted the dissolution rates to keep predicted pore water concentrations in line with the observed concentrations.

The values for the opal dissolution rate found in literature all concerned first order rates for marine systems. For comparison with ECO’s calibrated rate (Eq. C.8) we re-calculated them for reference temperature 20 ^o^C (see above) and a dissolved silicate concentration 1.0 mg Si.L^−1^: for water k_od_ = 0.000625–0.005 (g Si.m^−3^)^−1^.d^−1^, for sediment k_od_ = 0.0005–0.00048 (g Si.m^−3^)^−1^.d^−1^ [8], [24], [37]. ECO’s rate calibrated for fresh sediment is significantly lower. The equilibrium dissolved concentration Si_e_ in ECO followed from maximum observed pore water concentrations in the sediment of Dutch shallow lakes [64].

Several publications provide data on the oxidation rate of sulphide (Eq. D.1): for marine water k_so_ = 51.3 (mg O_2_.L^−1^)^−1^.d^−1^, for marine sediment k_so_ = 2.06–23.0 (mg O_2_.L^−1^)^−1^.d^−1^ [1], [2], [17], [22], [29], [30], [63], for fresh sediment k_so_ = 85.0 (mg O_2_.L^−1^)^−1^.d^−1^ [20]. Because of insufficient data for fresh sediment we selected ECO’s oxidation rate of sulphide as an approximate average of the range for marine sediment. We gave the precipitation and dissolution rates of particulate sulphide k_sp_ and k_sd_ in ECO (Eqs. D.2–4) high values to ensure that predicted dissolved sulphide stays close to the equilibrium concentration Cs_e_. We derived the equilibrium constants for the sulphide speciation in solution from Stumm & Morgan [44].

The values for the methane oxidation rates (Eqs. D.5–6) found in literature all originate from Boudreau [15]: for marine and fresh sediment k_moo_ = 8.56 (mg O_2_.L^−1^)^−1^.d^−1^ and k_mos_ = 8.77 10^−6^ (mg S.L^−1^)^−1^.d^−1^ [17], [20], [30]. Luff & Moll [17] used the same high rate for both. We adjusted ECO’s values to obtain very low methane concentrations in an oxic water column.

Low values for the net settling velocities of particulate matter in several shallow lakes in the Netherlands that are exposed to rather continuous windy conditions resulted from unpublished model calibrations. However, the net settling velocities v_x_ (Eq. F.1) re-calibrated by us for very shallow Lake Veluwe are slightly larger than reported previously [26], respectively 0.23 m/day and 0.115 m/day for the deep and the shallow compartments. The difference is justified by less resuspension occurring in the deep compartment.

We derived the seepage and infiltration velocities (Eq. F.1) from an unpublished hydrological study for Lake Veluwe [26], [60]. v_spg_ is −0.015 and 0.0057 m.d^−1^ in the relatively deep and shallow compartments, respectively.

The dispersion coefficients for solutes and particulates in the sediment D_s_ and D_p_ (Eqs. F.4–5) have maximal values at the sediment-water interface and decrease exponentially with sediment depth. The present values in ECO for Lake Veluwe are based on values reported by Smits & Van der Molen [25]. D_s_ at the sediment-water interface varies between 1.78×10^−4^ m^2^.d^−1^ (January-February) and 7.73×10^−4^ m^2^.d^−1^ (June-September), whereas the average molecular diffusion coefficient corrected for tortuosity (porosity^2^) is 0.75×10^−4^×0.55^2^ = 2.27×10^−5^ m^2^.d^−1^. The exponential decrease implies a decrease to 10% at a sediment depth of 5 cm, below which the coefficient is almost equal to the corrected molecular diffusion coefficient. These values agree with values found in the literature that imply a maximal bioirrigation enhancement factor 5 [25], [29], [36]. The bioturbation dispersion coefficient D_p_ near the sediment-water interface in ECO varies linearly between 2.8×10^−6^ m^2^.d^−1^ (June-September) and 2.8×10^−7^ m^2^.d^−1^ (January-February). Exponential decrease implies that D_p_ decreases to 10% at a sediment depth of 4 cm and is virtually equal to zero at a depth of 10 cm. This agrees with values reported in literature for other models: D_p_ = 0–2.0 10^−6^ m^2^.d^−1^ (seasonal variation fresh water system; [20], [25]), D_p_ = 2.36–8.0 10^−6^ m^2^.d^−1^ (marine water; [17], [29], [38]).

The extinction coefficients for particulate substances in ECO (Eqs. H.1–4) were calibrated for several lakes in the Netherlands and agree with values reported elsewhere. Pätsch & Kühn [4] provide es_im1/pom_ = 0.06 (m.g.m^−3^)^−1^ and e_b_ = 0.09 m^−1^. Blauw et al. [24] report es_poc_ = 0.1 (m.g.m^−3^)^−1^, es_im1_ = 0.025 (m.g.m^−3^)^−1^ and e_b_ = 0.09 m^−1^. We calibrated the extinction coefficient of dissolved organic matter (DOC) with regard to light limitation and secchi depth.

## Calibration Simulation Results, Model Performance

### Water Quality and Phytoplankton

**Figure 3:**
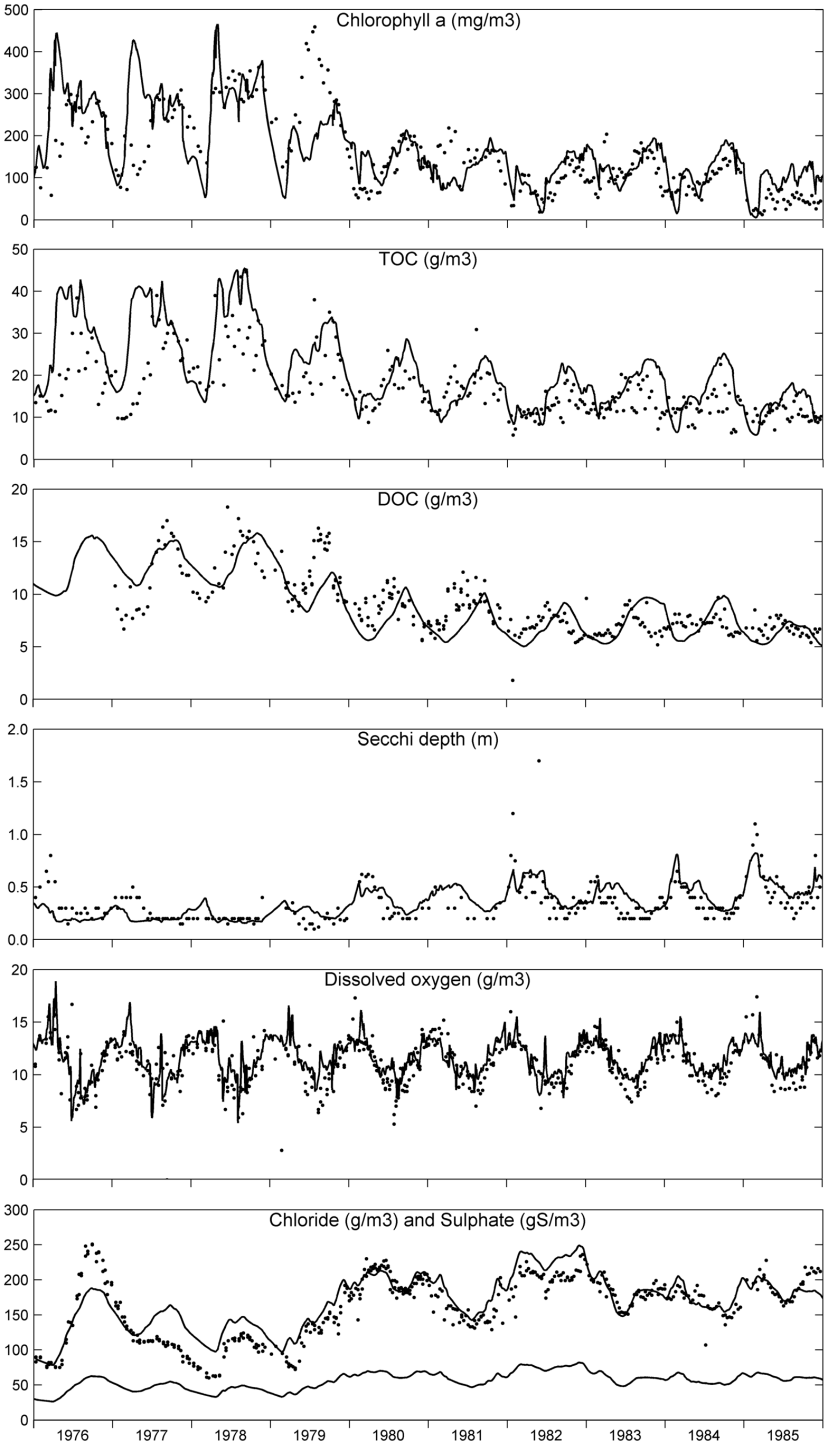
Simulated (lines) and observed (dots) water quality parameters in Lake Veluwe. The parameters represent the concentrations of chlorophyll-a, total organic carbon and dissolved organic carbon, the secchi depth, and the concentrations of dissolved oxygen and chloride (paired with sulphate, lower line) in the water column of Lake Veluwe for the period 1976–1985.

**Figure 4:**
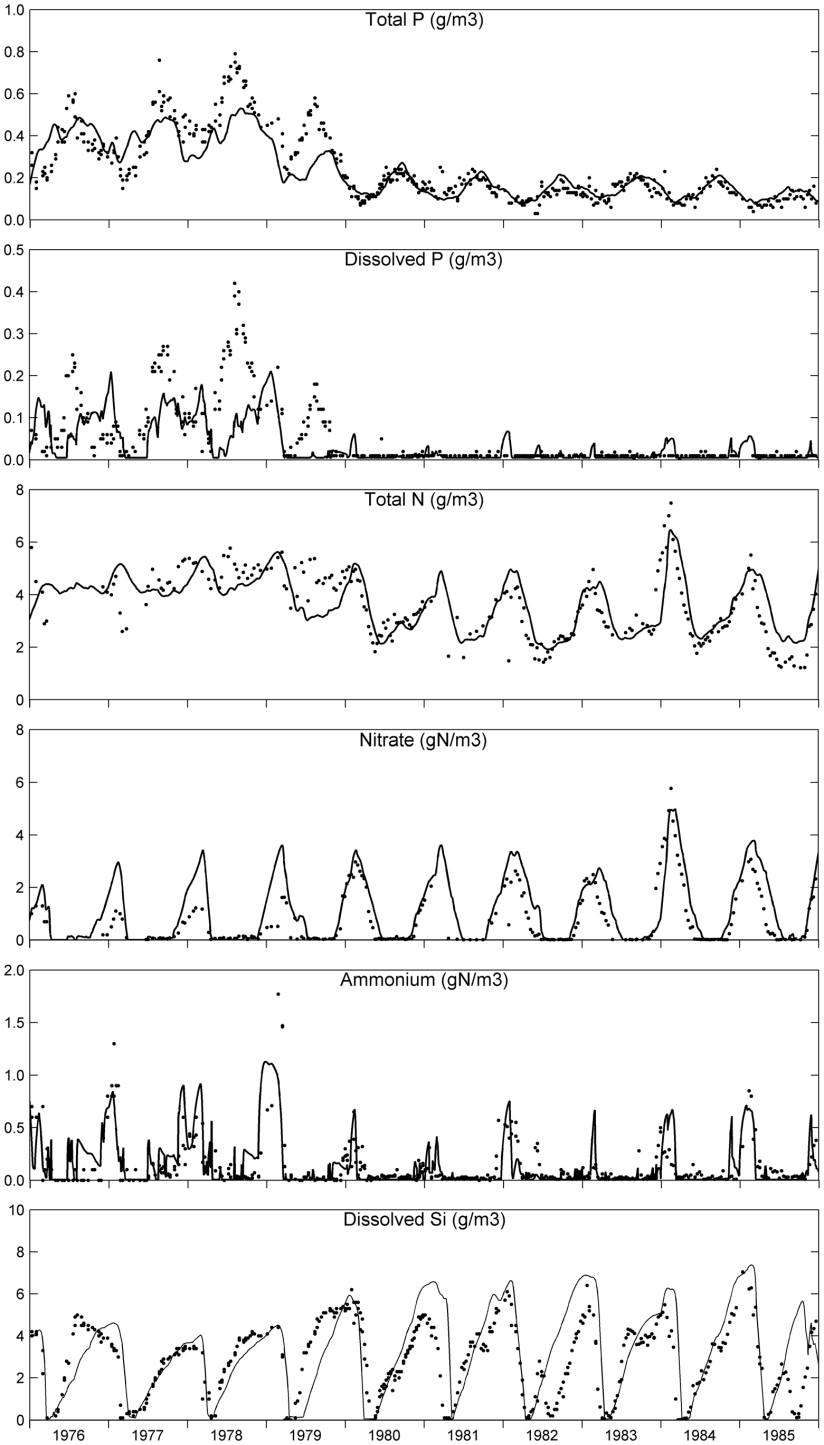
Simulated (lines) and observed (dots) nutrient parameters in Lake Veluwe. The parameters represent the concentrations of total phosphorus, inorganic dissolved phosphorus, total nitrogen, nitrate, ammonium and dissolved silicate in the water column of Lake Veluwe for the period 1976–1985.

The simulated and observed concentrations indicated in Figure 3 (phytoplankton, organic matter, secchi depth, oxygen, chloride and sulphate) and Figure 4 (nutrients) represent the average water quality of the deep and shallow compartments. Substantially more parameters are shown than for the DBS Lake Veluwe model [26], which is possible due to ECO’s comprehensiveness. The observed concentrations represent the data collected for two sampling locations, one in each compartment.

The results for chloride show that the water balances imposed on the model for 1978–1985 are quite accurate. The rather large differences of simulated and observed chloride suggest that the water balances for 1976 and 1977 are not very accurate, which has a bearing on the accuracy of the nutrient loads. No data are available for sulphate in Lake Veluwe, but the predicted ratio of sulphate and chloride is in line with data for other similar lakes in the Netherlands.

Simulated and observed chlorophyll-a match quite well for most of the simulated period. The steep transition caused by the reduction of the nutrient loads and flushing is reproduced by the model. However, chlorophyll-a is overpredicted for the spring peaks in 1976–78, whereas the summer peak in 1979 and the spring peak in 1981 are underpredicted. Both underpredictions are essentially caused by P-limitation as appears from the lack of dissolved phosphate in the water column during these periods. This points at temporary underprediction of the internal P-load (P-return flux from the sediment) and/or the temporary underestimation of the external P-load. Structural overprediction is found for 1985, most probably due to the absence in the model of macrophytes and microphytobenthos that began to re-appear in Lake Veluwe in 1985 due to its much enhanced transparency [60], [61]. Nutrient uptake by macrophytes and microphytobenthos decreases the total nitrogen and total phosphorus concentrations in the lake, so that less nutrients are available to phytoplankton and phytoplankton biomass (chlorophyll-a) is reduced.

**Figure 5:**
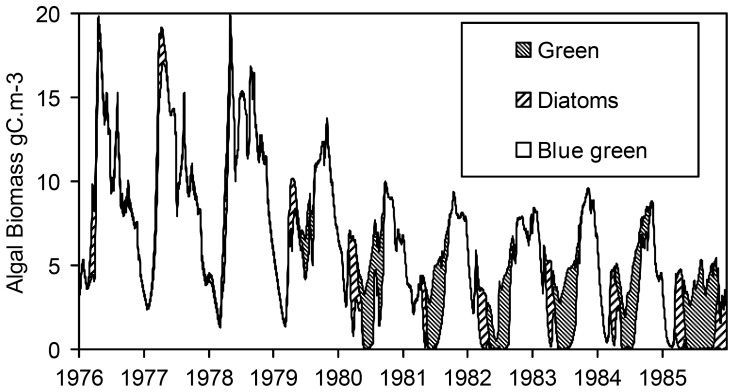
Simulated phytoplankton species composition in Lake Veluwe for the period 1976–1985.

The simulated phytoplankton species composition (Fig. 5) is very similar to the composition simulated with the model described by Van der Molen et al. [26], which also contained BLOOM. The dominance of cyanobacteria(mainly *Oscillatoria agardhii*) in 1976–1979 changes into dominance of diatoms in spring and of green algae in most of the summer as of 1980. Cyanobacteria are almost entirely absent as of 1985. Van der Molen et al. [26] stipulated that this agreed with observed species composition.

Total organic carbon (TOC) is the sum of the carbon stored in algae biomass, the particulate detritus fractions (POC1–4) and dissolved organic carbon (DOC). The simulation results and the observed concentrations for TOC, DOC and dissolved oxygen agree quite well, which supports the claim that primary production by algae is simulated rather accurately by the model. Over- and underpredictions are consistent with over- and underpredictions of chlorophyll-a. The model reproduces observed low secchi depth in 1976–1979 with deviations that are also consistent with this. Simulated secchi depth agrees quite well with the observed increase of the secchi depth in 1980–1985. The light limitation of phytoplankton that occurred in the deep compartment of Lake Veluwe in 1976–1978 was replaced by P-limitation except for periods in winter.

Whereas simulated total phosphorus agrees well with measured total phosphorus as of 1980, summer peaks are underpredicted for 1976–1979. This may be due to underpredicted P-return fluxes from the sediment, as dissolved phosphate is similarly underpredicted, but the phosphorus loads may have been underestimated too. Possibly not enough adsorbed phosphate is stored in the top sediment in the model, or adsorbed phosphate is not released fast enough during the summers of 1977–1979. We could not tune the adsorption related coefficients of the model such that underprediction in 1977–1979 was removed and that overprediction as of 1980 was prevented at the same time. Enhanced precipitation of phosphate minerals in the sediment, the apatite-like mineral in particular, was needed to keep dissolved phosphate as low as observed and to bring about sufficient P-limitation of the algae. Trial simulations showed overprediction of dissolved phosphate as of 1980 when we decreased the ratio of apatite over vivianite precipitation r_av_ or the precipitation rate k_vp_. Although no specific evidence exists for the presence of an apatite-like mineral in the sediment of Lake Veluwe, the assumption of the formation of an authigenic apatite-like mineral is justifiable by the presence of calcite in this sediment and the possible co-precipitation of apatite with calcite.

Some of the summer dips and the winter peaks of total nitrogen are overpredicted by the model. The overprediction of winter peaks shows in nitrate as well. Nitrogen return fluxes might be overestimated due to insufficient denitrification, but nitrogen loads may have been overestimated too. Trial simulations pointed out that enhanced nitrification and denitrification caused underprediction of summer chlorophyll-a in 1977–1979, because phytoplankton is N-limited in summer and most of the autumn.

The observed dips and peaks of dissolved silicate as resulting from the spring diatom blooms are reproduced by the model, which supports the validity of the predicted algae species succession.

### Sediment and Pore Water Quality

**Figure 6:**
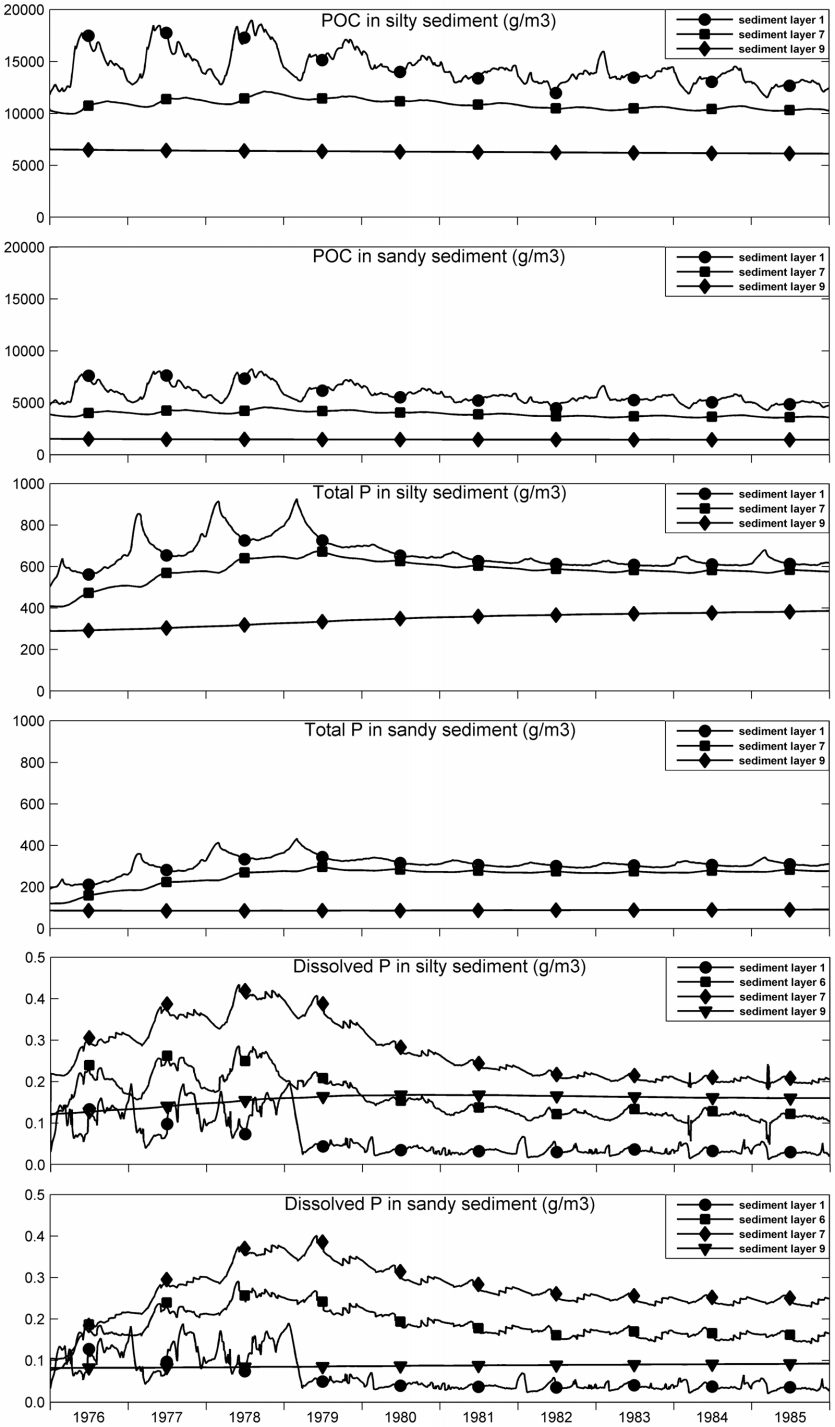
Simulated sediment quality parameters in Lake Veluwe. The parameters represent the bulk concentrations of particulate organic carbon and total phosphorus, and the pore water concentration of dissolved inorganic phosphorus in the sediment of Lake Veluwe for the period 1976–1985. The silty sediment is in the “deep” compartment, the sandy sediment is in the shallow compartment.

Simulated concentrations of POC, total P, and dissolved inorganic P in the sediment of Lake Veluwe show accumulation in the hypertrophic period 1976–1979 (Fig. 6). Whereas ECO provides much more vertical detail than the SWITCH module of DBS [25], a selection of the results for three or four out of ten layers is presented. After the large decrease of the P-load on the lake in 1979, this is followed by a decline of concentrations in the sediment during 1980–1985, which for the top sediment of the deep compartment is mainly due to the bioturbation of particulate phosphate into deeper sediment and the loss of dissolved phosphate into the groundwater below the “deep” compartment (see also [26], [60]). The decline of total phosphorus in the top sediment of the shallow compartment is much weaker due to the import of phosphorus from upwelling groundwater. As can been seen from Table 6, the simulated total phosphorus and organic carbon contents in the sediment agree quite well with observed contents, that were derived from Brinkman & Van Raaphorst [59] (see [25]). We derived simulated contents from bulk concentrations by division by (1-porosity) and sediment density (2.6 kg.L^−1^). The concentrations predicted by the model for the individual phosphorus components point out that at the end of the 1976–1985 simulation approximately 40% of total P in the sediment is stored in an apatite-like mineral in both compartments. Observed dissolved phosphorus in pore water of the upper 10 cm sediment for 1983 shows rather irregular patterns, but a distinct maximum occurred between 1–2 cm sediment depth and minimum values were found below 3 cm depth [59]. Concentrations are higher in summer than in spring. These trends are reproduced by the model. The mean observed concentration is 0.198 g P.m^−3^ (st. dev. = 0.192 g P.m^−3^, n = 94). The simulated average dissolved inorganic phosphorus concentrations (PO4) in the top 10 cm of sediment for 1983 vary around 0.17 g P.m^−3^ in the deep compartment, and around 0.14 g P.m^−3^ in the shallow compartment. Simulated dissolved organic phosphorus varies around 0.035 g P.m^−3^.

**Table 6:** Simulated and observed total phosphorus and organic carbon contents (g.kg DM^−1^) in sediment.

parameter	1979–81			1982		
	mean	st.dev.	n	mean	st.dev.	n
upper 10 cm of silty sediment in the deep compartment						
observed TP	0.54	0.18	21	0.59	0.095	12
simulated TP	0.59			0.59		
observed OC	11.6	5.9	18	9.1	5.2	19
simulated OC	10.4			10.0		
upper 10 cm of sandy sediment in the shallow compartment						
observed TP	0.094	0.048	10	0.078	0.033	10
simulated TP	0.10			0.10		
observed OC	1.60	1.3	18	1.60	1.1	19
simulated OC	1.57			1.49		

The observation data were reported in Smits & Van der Molen (1993).

**Figure 7:**
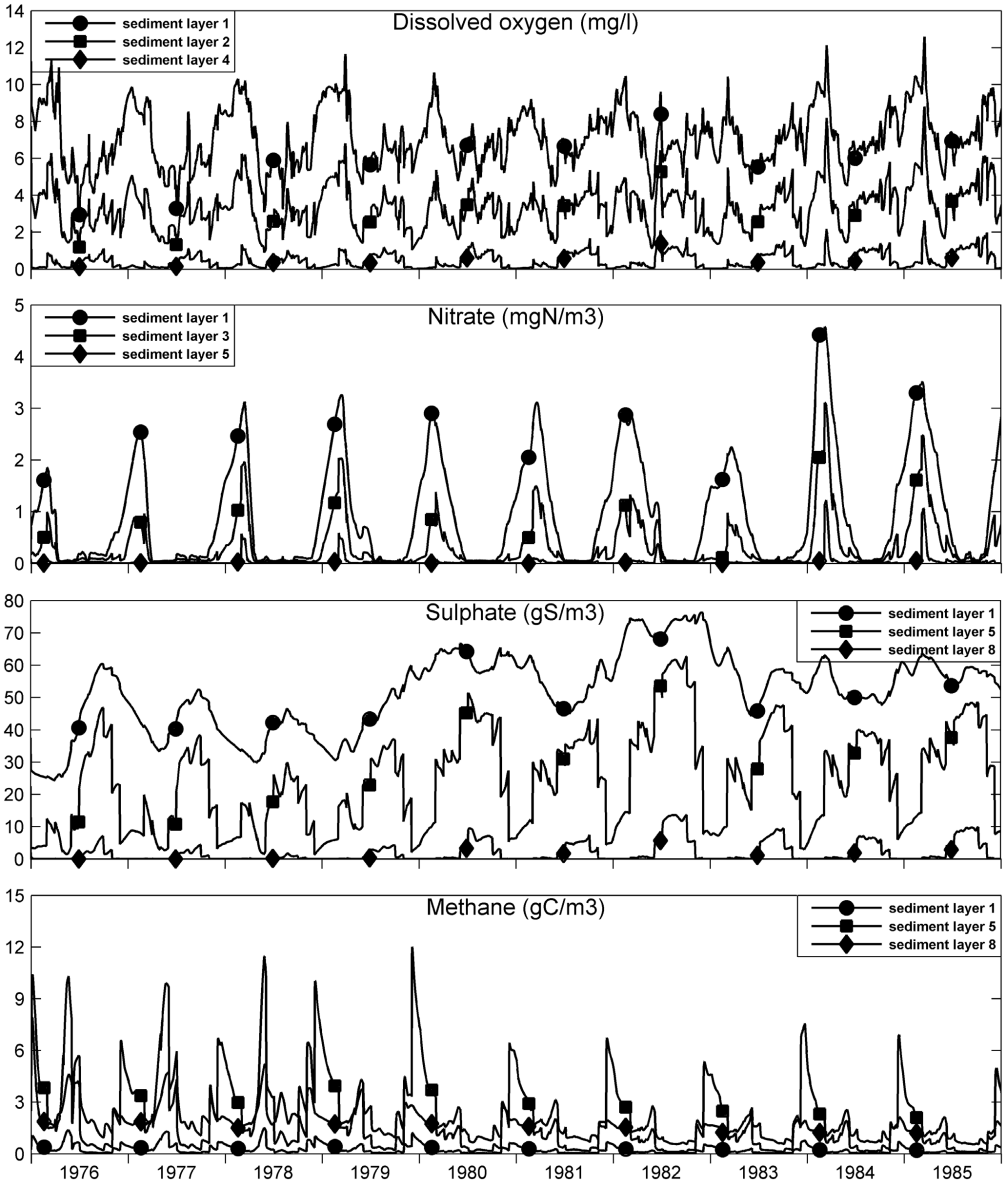
Simulated pore water quality parameters in Lake Veluwe. The parameters represent the pore water concentrations of dissolved oxygen, nitrate, sulphate and methane in the sandy sediment of Lake Veluwe for the period 1976–1985.

The simulated pore water concentrations of the electron-acceptors dissolved oxygen, nitrate, and sulphate in the sediment of Lake Veluwe clearly show seasonal variation (Fig. 7; sandy sediment). The oxic layer is much thinner in the summer half year than in the winter half year, when the decomposition of organic matter is slow. During the summers of 1976–78 the oxic top layer becomes approximately 1 mm thick in the model, which implies the collapse of the adsorption capacity for phosphate and the strong increase of dissolved phosphorus. A structural change occurs in 1979, when the penetration of dissolved oxygen, nitrate and sulphate into the sediment in summer increases and methane decreases because of highly reduced detritus settling on the sediment, which leads to the enhanced adsorption of phosphate and strongly decreased dissolved phosphorus.

**Figure 8:**
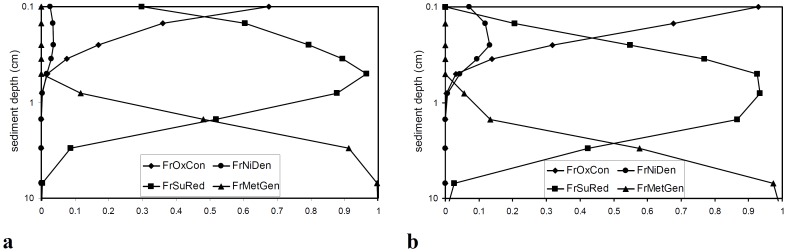
Simulated fractional contributions of electron acceptors to the mineralization of organic matter. The contributions are delivered by oxygen consumption, denitrification, sulphate reduction and methanogenesis in the sandy sediment of Lake Veluwe for 1 July 1978 (a) and 1 July 1982 (b).

Typical summer situations for the simulated fractional contributions of oxygen consumption, denitrification, sulphate reduction and methanogenesis to the mineralization of organic matter in the sediment of Lake Veluwe are shown in Figure 8 for sandy sediment. The contributions are partially overlapping, with a near 100% contribution of oxygen in the upper sediment layer (1 mm) and a near 100% contribution of methanogenesis in the lower sediment layer (4–10 cm). The contributions of oxygen consumption and denitrification increase significantly after 1979, whereas the contribution of sulphate reduction decreases strongly.

### Mass Balances and Return Fluxes

**Figure 9:**
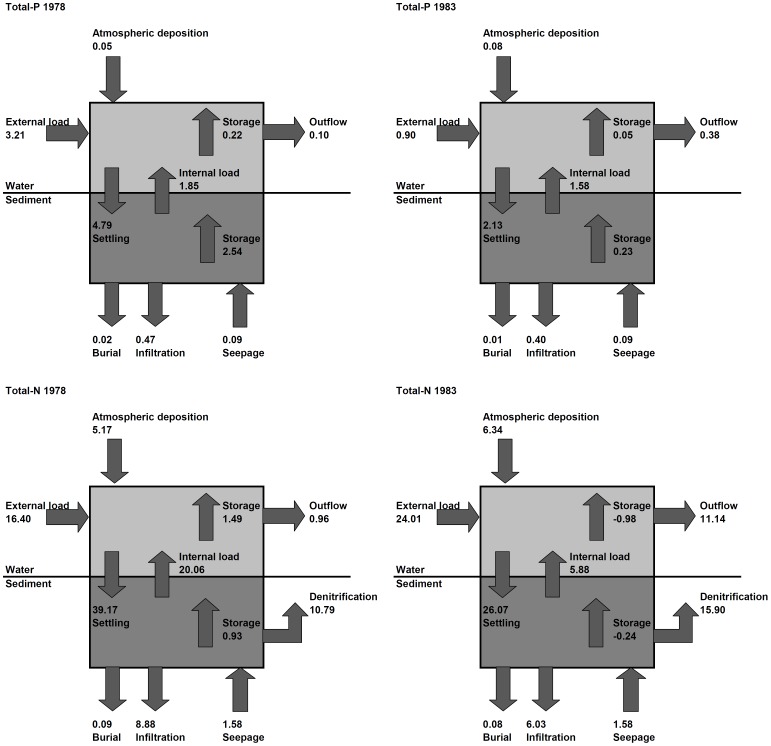
Mass balances of phosphorus and nitrogen for Lake Veluwe. The mass balances are derived from the process mass fluxes as simulated for 1978 and 1983. Fluxes are in g P.m^−2^.yr^−1^ or g N.m^−2^.yr^−1^.

The mass balances for phosphorus and nitrogen of Lake Veluwe for 1978 and 1983 that result from the model are displayed in Figure 9. The total external load is the sum of the external load (from surface water and point sources), the seepage load and atmospheric deposition. The internal load is the diffusive return flux of ammonium and nitrate or of phosphate from the sediment to the overlying water. Outflow is the net transport of nutrients to adjacent Lake Dronten north east of Lake Veluwe. Infiltration in the “deep” compartment and seepage in the “shallow” compartment represent the transport to and from groundwater. Burial is the removal of substances into the sediment below the simulated layers resulting from the net settling of sediment. Storage is the difference between initial and final composition, which in a “dynamic steady state” should be equal to zero. Positive storage and burial imply accumulation in the sediment.

The mass balance data produced by ECO are more detailed compared to the DBS data [26], and allow for a more detailed assessment of the various nutrient fluxes and removal processes. As appears from Figure 9, from 1978 to 1983 the total external load of phosphorus dropped 68%, whereas the total external load of nitrogen increased 38% due to flushing with nitrogen rich water. The internal load of phosphorus decreased only 15%, and becomes much larger than the external load in 1983, despite the enhanced adsorption of phosphorus in the top sediment due to the strongly reduced input of detritus. This shows that internal eutrophication can continue years after an external load reduction. The average simulated summer half year return flux of dissolved phosphate for 1978 is 11.6 g P.m^2^.d^−1^ for the silty sediment and 7.5 g P.m^2^.d^−1^ for the sandy sediment. For 1983 these fluxes are 6.2 g P.m^2^.d^−1^ and 5.9 g P.m^2^.d^−1^, respectively. The mean phosphate return flux observed in-situ with a benthic chamber in the summer half year of 1983 is 4 mg P.m^2^.d^−1^ (s.d. = 5, n = 2) for the silty sediment [59], [25]. The predicted phosphate return fluxes for 1983 have similar magnitudes as the return fluxes observed for the silty sediment.

The internal load of nitrogen is 87% of the total external load in 1978. It becomes much smaller and drops to only 18% of the total external load in 1983 because coupled nitrification-denitrification is enhanced strongly in a more oxidizing top sediment layer. The denitrification flux becomes larger but remains approximately 50% of the total external load at a strongly reduced residence time.

In 1978 the net outflow of phosphorus is only 3% of the total external phosphorus load, the accumulation in the sediment 76%, and loss by infiltration into groundwater 14%. Due to flushing the net outflow increases to 36% of the total external load in 1983. The accumulation is down to 22%, whereas infiltration increases to 37%. Similarly the net outflow of nitrogen increases from 4% to 35% of the total external loads. The loss of nitrogen by infiltration goes down from 38% to 19%, whereas accumulation is unimportant. The large removal fluxes of nutrients by infiltration indicate its importance for the oligotrophication of Lake Veluwe. With respect to phosphorus, flushing was equally important.

## Discussion and Conclusions

The present article describes the content and the calibration of the eutrophication model ECO as based on DELWAQ that combines a number of significant modeling innovations. Compared to other eutrophication models, DBS [26] included, ECO contains the following innovations:

- Comprehensive water and sediment quality are simulated in a generic way on the basis of a computational grid covering both water column and sediment bed. Process formulations are deterministic and identical for water column and sediment bed. Chemical conditions determine how formulations turn out locally.
- Process formulations are based on state-of-the-art process kinetics. Simplified formulations are used only for the formation of phosphate minerals.
- Concentration gradients and nutrient return fluxes across the sediment-water interface are simulated dynamically on the basis of comprehensive sediment diagenesis taking into account the dominant electron-acceptors (dissolved oxygen, nitrate, sulphate, carbon monoxide) and redox processes.
- Nitrification, denitrification and phosphate retention in the sediment proceed according to conditions in the sediment.
- Distinction is made between redox sensitive vivianite-P and stable apatite-P.
- The adsorption capacity is deduced from the reactive iron content of the sediment corrected for the oxidized iron(III) fraction.
- The mass balances for organic carbon, nutrients (N, P, Si), sulphur and oxygen are fully closed for water and sediment. All relevant organic and inorganic components are included.
- Covering the entire organic matter cycle in water and sediment, detrital organic matter is simulated with four particulate fractions and one dissolved fraction, having different decomposition rates, the one fraction being produced from the other.
- Light extinction is calculated from contributions of all major components, including dissolved organic matter.
- The 1D, 2D or 3D computational grid for water and sediment is defined by input. Number and thickness of layers are only limited by the maximum acceptable computational burden.

The process coefficients that resulted from the calibration of the Lake Veluwe model are in line with the ranges of values that were reported for other models. The simulation results show ECO’s capacity to simulate water and sediment quality and sediment-water exchange fluxes dynamically and quite accurately. The results underline the importance of redox processes and phosphate speciation for the nutrient return fluxes.

Differences between simulated and observed water quality mainly concern the timing and the magnitude of concentration peaks and dips. Parts of these differences are due to inevitable inaccuracies in model forcing, in water and nutrient loads and in suspended inorganic sediment in particular. The tendency of the model to underestimate the variability of concentrations is partially caused by the application of net settling in stead of alternating settling and resuspension. However, the stochastic patchiness of lake water quality cannot be reproduced by a model with two compartments and forcing that is averaged over time and space.

The underprediction of the nutrient return fluxes in Lake Veluwe in the summers of 1976–1979 may be due to the set-up of the present model. The simulation shows that the oxic top layer of the sediment can become very thin in the model, but can never disappear when the overlying water still contains substantial concentrations of dissolved oxygen. Near bottom thermal stratification may have occurred in the “deep” compartment during summer periods with low wind speed, leading to near zero oxygen concentrations just above the sediment, and to stronger chemical reduction, further loss of adsorption capacity and reduced nitrification-denitrification in the upper sediment layer. The present model with fully mixed water column cannot generate these low oxygen concentrations, and therefore tends to underpredict the phosphate and ammonium return fluxes in summer. The near bottom anoxic conditions due to stratification might be reproduced with a 3D model based on stratified water flow.

The overprediction of nutrients and phytoplankton for 1985 is probably due to the absence of submerged macrophytes in the model. These macrophytes that re-appeared in substantial quantities in Lake Veluwe as of 1985 store significant amounts of nutrients that are therefore not available for phytoplankton. The growth of microphytobenthos also not included in the model may have contributed too to observed lower nutrient concentrations in the water column. The overprediction of phytoplankton in Lake Veluwe for 1985 and years after (not shown here) shows that to more accurately simulate phytoplankton and nutrients in shallow lakes with nutrient limitation and a large population of macrophytes these macrophytes and possibly also microphytobenthos should be included in a eutrophication model.

The simulation of the water quality of Lake Veluwe with comprehensive ECO provides additional insight in how lakes may respond to nutrient load reduction and flushing. The following conclusions are drawn additional to the conclusions resulting from previous modeling of Lake Veluwe [25], [26]. In the model a 68% phosphorus load reduction in 1979 resulted in a 32% reduction of the summer half year average return flux of phosphate from the sediment in Lake Veluwe four years after the load reduction. This shows that internal eutrophication can continue years after an external load reduction. Although the oligotrophication of Lake Veluwe resulted primarily from the load reduction, the removal of nutrients by infiltration and flushing contributed substantially. Moreover, the observed decrease of total phosphorus in the lake could only be reproduced by the model by including the precipitation of phosphate in an apatite-like mineral in the sediment, that binds phosphate more or less permanently. This suggests that authigenic formation of a stable apatite like mineral can contribute significantly to oligotrophication of a lake after a nutrient load reduction. The model also shows that the removal of nitrogen in the sediment by coupled nitrification-denitrification may be enhanced when the thickness of the oxidizing top sediment layer increases due to reduced detritus settling.
